# Effect of Cellulose Nanocrystals Nanofiller on the Structure and Sorption Properties of Carboxymethyl Cellulose–Glycerol–Cellulose Nanocrystals Nanocomposite Systems

**DOI:** 10.3390/ma13132900

**Published:** 2020-06-28

**Authors:** Maria-Cristina Popescu, Bianca-Ioana Dogaru, Carmen-Mihaela Popescu

**Affiliations:** Petru Poni Institute of Macromolecular Chemistry of the Romanian Academy, 700486 Iasi, Romania; cpopescu@icmpp.ro (M.-C.P.); dogaru.bianca@icmpp.ro (B.-I.D.)

**Keywords:** cellulose nanocrystals, carboxymethyl cellulose, structural features, sorption properties, interactions

## Abstract

Biobased materials present a great interest due to their properties and biodegradability. Cellulose nanocrystals (CNC) nanofiller, in various amounts, was incorporated into a carboxymethyl cellulose (CMC)–glycerol (G) matrix in order to obtain nanocomposite systems with improved properties. The effect of the nanofiller on the structural features was investigated by Fourier transform infrared (FT-IR) spectroscopy, principal component analysis (PCA), two-dimensional correlation spectroscopy (2D-COS), and X-ray diffraction, while the sorption properties were evaluated by water vapor isotherms using the gravimetric method coupled with infrared spectroscopy. We observed the presence of the interactions taking place between the CMC-G and CNC involving the hydroxyl and carboxylate groups, which decreased the number of water sorption sites. Following this, the moisture content in the nanocomposite films decreased with the increase in the amount of CNC. Moreover, the bands associated to water molecules presented different wavenumber values separated for CMC-G and CNC components.

## 1. Introduction

Cellulose, one of the most abundant natural biopolymers, is biosynthesized by nature with an amount of 10^11^–10^12^ tons/annum. The repetitive monomeric unit of cellulose is D-glucose [anhydroglucose unit (AGU)], which links successively through a β-1,4 bond between C1 and C4 of adjacent units [1]. It forms strong intermolecular hydrogen bonds, leading to regions that are both crystalline and amorphous and form microfibrils and fibers. Cellulose was widely used as a raw material in several applications, and it was further extended to its derivatives, which are known for their wide use in different applications, e.g., carboxymethyl cellulose, methylcellulose, cellulose nitrate, ethyl cellulose, hydroxypropyl cellulose, hydroxyethyl cellulose, cellulose acetate, etc. [2]. By using biopolymers such as carboxymethyl cellulose, some of the environmental problems given by the use of synthetic polymers can be eliminated. 

Carboxymethyl cellulose (CMC) a well-known cellulose derivative, is a high molecular weight water-soluble anionic polysaccharide obtained by treating cellulose with chloroacetic acid. CMC is generally recognized as a safe (GRAS) material, so it has been used in the food industry as a thickener or stabilizer [3] and pharmacy as a hydrogel for wound dressing and drug delivery [4]; it has good film-forming properties, high optical transparency, and chemical stability, and it is also biodegradable, non-toxic, biocompatible, tasteless, and odorless [5]. As a result of CMC’s drawbacks, especially its highly hydrophilic nature and poor thermomechanical properties, its use in some applications is limited [6]. These “problems” can be solved by adding nanosized fillers into the polymer matrix [7], and one would be cellulose nanocrystals (CNCs). They have a similar backbone structure; therefore, they can form nanocomposite films with a high degree of compatibility.

Nanocelluloses are a relatively new class of bionanomaterials obtained by acid hydrolysis with sulfuric acid when cellulose nanocrystals (CNCs) [7] are synthetized. They have impressive properties, being non-toxic, biodegradable, and renewable, having good mechanical properties and a high surface area. The good strength properties (the Young’s modulus is estimated to be up to 167 GPa and the tensile strength of the crystal structure is around 0.8–10 GPa [8]), the thermal stability of the CNC, and high modulus of elasticity make them suitable for use as reinforcement in bionanocomposites [9]. Cellulose nanocrystals, due to their structure, are capable of forming many hydrogen bonds with the matrix [10], and their homogeneous distribution within a matrix and strong interactions result in clear improvements in the vapor barrier properties, as well as thermal and mechanical properties of the nanocomposites [11,12]. Adding CNC in other polysaccharides, such as hemicellulose, chitosan, and starch [10], the water vapor barrier can be improved due to the increasing of the crystallinity of the matrix [9]. El Miri et al. [13] prepared composite materials blending CMC, starch, and CNC, and they observed that the high number of functional groups present in CNC and biopolymer matrices improved the interfacial interactions between the biopolymer matrix and CNC. Many biodegradable composite materials have been prepared from CMC or combination of CMC with other carbohydrates, proteins, lipids, and different reinforcing agents, resulting in composite materials with enhanced mechanical, thermal, and barrier characteristics in comparison to pure biopolymers [14]. 

The objective of the present work was to develop nanocomposite films combining CMC (as matrix), CNC (as nanofiller), and glycerol (as plasticizer). Further, the influence of the nanofiller on the structure and sorption properties of these films was evaluated by infrared spectroscopy, X-ray diffraction, and scanning electron microscopy. In order to be used as coating materials, the moisture sorption properties are important; therefore, water vapor sorption coupled with infrared spectroscopy was performed, and the water uptake properties were evaluated.

## 2. Materials and Methods

### 2.1. Materials

The materials used in this study were carboxymethyl cellulose sodium salt in the form of white powder (M.W.: 90.000 g/mol) (CMC), glycerol (N99.5%) (G) purchased from Sigma Aldrich (Sigma Aldrich, St. Louis, MO, USA), cellulose nanocrystals (CNC) purchased from Melodea (Melodea Bio Based Solutions, Rehovot, Israel), and double-distilled water.

### 2.2. Preparation

For the preparation of the nanocomposite systems, a solution of 3 wt % CMC was obtained by its solubilization in distilled water at 65 °C, and 1500 rpm, for 1 h. In the obtained CMC solution, different amounts of CNCs were added separately (see Table 1). After homogenization, in each solution was added glycerol (30 wt % based on the total weight of dry mass of CMC and CNC components). Further, the mixture was homogenized using a magnetic stirring plate for 30 min at 65 °C and 1500 rpm and then at 10,000 rpm for 10 min with an ultraturax, to guarantee a better homogenization of the components in the solution.

**Figure 1:**
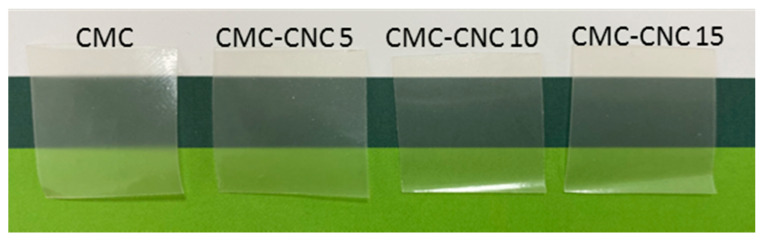
Visual aspect of CMC-G and CMC-CNC films.

The resulted formulations were transferred to Petri dishes and dried at 45 °C for about 24 h (until the films were dried and no variation in the mass was recorded). After drying, the films were of about 0.15 ± 0.015 mm thickness. The prepared films are transparent, as can be seen in Figure 1.

### 2.3. Methods

#### 2.3.1. Infrared Spectroscopy

The Fourier transform infrared (FT-IR) spectra of the films were recorded Bruker ALPHA FT-IR spectrometer (Bruker, Karlsruhe, Germany) in attenuated total reflection (ATR) mode (equipped with a Diamond crystal) at 4 cm^−1^ resolution and in the 4000–500 cm^−1^ spectral range. For each sample, five spectra were recorded, and the evaluations were made using the average spectrum. Processing of the spectra was done in OPUS 7.5 program and Grams 9.1 (ThermoFisher Scientific, Waltham, MA, USA). PCA was performed on pre-processed spectra in OPUS 7.5 program (Bruker, Karlsruhe, Germany), while 2D-COS was performed in Matlab R2018b (The MathWorks, Inc., Natick, MA, USA).

#### 2.3.2. X-Ray Diffraction

The diffractograms were recorded on a Diffractometer D8 ADVANCE (Bruker AXS, Germany), using the CuKα radiation (λ = 0.1541 nm). The conditions were: 40 kV and 30 mA, 2 s/step, and 0.02 °/step, 10–40 2θ degrees range and room temperature. 

To assess the crystallinity degree of the studied samples, as well as the crystallite size of the (200) crystallographic plane and interplanar spacing, the diffractograms were deconvoluted with mixed Gaussian–Lorentzian profiles (for crystalline regions) and with Voight profile (for the amorphous background) [15,16]. These types of profiles proved to be a good approach, the reduced chi-squared being less than 0.1. The degree of crystallinity was calculated according to Equation (1), which was proposed by Hermans and Weidinger (1948) and used after that by other researchers [15,16,17]. The crystallite size was calculated according to Scherrer Equation (2) [15,18], while the interplanar spacing was calculated by the Bragg Equation (3) [19]

CrI% = (A_cr_/A_t_) × 100, (1)

τ_200_ = (k × λ)/(β × cosθ), (2)

n × λ = 2d × sinθ,(3)

where A_cr_ is the crystalline area, A_t_ is the total area (crystalline and amorphous), τ is the crystallite size, k is the “shape factor” with a value of 0.94, λ is the wavelength (0.1542 nm), β is the peak width at half height (expressed in radians), θ is the position of the peak (half of the 2θ value), n is the order of reflection, and d is the interplanar spacing of the crystal [15,18,19].

#### 2.3.3. Scanning Electron Microscope (SEM)

The morphology of film samples was investigated using a Scanning Electron Microscope SEM EDAX Quanta 200 (FEI Company, Hillsboro, TX, USA), operating at 3 kV in high vacuum mode.

#### 2.3.4. Water Vapor Sorption

Previously, oven-dried samples were inserted in sealed containers with different relative humidity (RH) values (of 8.5%, 10%, 17%, 27%, 37%, 46%, 54%, 59%, 66%, 74%, 79%, and 83%) obtained using saturated salt solutions at 25 °C. To record the exact RH values, each container was equipped with a LogTag thermohydrometer. A sample was kept at a certain RH value until constant mass was recorded and then was transferred to the container with a higher RH value. The same procedure was applied for all the studied samples.

The moisture content (MC) was calculated by using the following equation:

MC% = [(W_1_ − W_0_)/W_0_] × 100, (4)

where W_1_ is the weight of the sample at a certain RH, while W_0_ is the oven-dried weight of the samples.

Immediately after weighting, for all the samples, the infrared spectra were recorded, and in this case, only the 3700–3000 cm^−1^ region was analyzed, which is assigned to different OH stretching vibrations and also to water sorption.

#### 2.3.5. Water Uptake

Oven-dried samples were placed in a desiccator at 57% RH (using Mg(NO_3_)_2_ saturated solution). The samples were removed from the medium and weighed periodically until a constant mass was observed. The water uptake (WU%) values were obtained by using the following equation:

WU% = [(M_t_ − M_i_)/M_i_] × 100, (5)

where M_t_ is the mass of the films recorded at a time *t*, and M_i_ is the dry mass of the sample.

## 3. Results and Discussions

### 3.1. Infrared Spectroscopy

FT-IR spectroscopy is a simple and useful technique to obtain rapid information concerning the structure, chemical changes, and interactions taking place in composite materials [20,21,22]. In the present study, the structural features of the CMC-CNC composites were evaluated. The films were prepared using CMC (as matrix) and CNCs (as nanofiller) in different amounts and glycerol (as plasticizer). To detect only the influence of the CNC on the final films, we compared the composite films with the CMC-G film (as reference).

Generally, the carbohydrate spectra are complex, being divided in two important regions, i.e., 3720–2700 cm^−1^ and 1750–870 cm^−1^. The characteristic bands of the components and their modifications due to the presence of the reinforcing agents can be better observed in the second derivative spectra because this method can enable distinguishing the individual overlapping bands.

**Figure 2:**
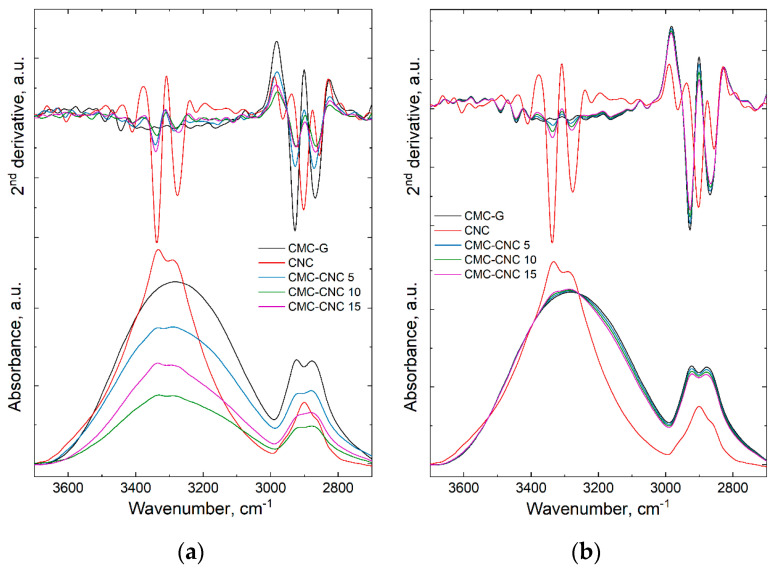
Experimental (**a**) and theoretic (**b**) infrared spectra and their second derivatives in the 3700–2700 cm^−1^ region of the components and nanocomposites.

The first region (Figure 2a) is useful to elucidate the H-bonding patterns, each distinct OH groups giving a specific stretching vibration at a certain frequency. Here, it is represented by a large band with a maximum at 3278 cm^−1^ for CMC-G film and by a large band with two maxima at 3333 and 3286 cm^−1^ for CNC. The addition of CNC into the CMC-G matrix can be observed in the spectra as a combination between the two spectra with variable intensities. Further, in the same region, two other bands at 2921 and 2875 cm^−1^ are observed for the CMC-G film, while only one maximum at 2902 cm^−1^ with a shoulder at 2866 cm^−1^ is observed for the CNC film. These bands are assigned to the methyl and methylene groups’ stretching vibration. 

To identify the presence of the interactions between the components, the theoretic spectra (Figure 2b) were calculated by using the CMC-G and CNC component spectra and the additivity law, based on the FT-IR spectra of pure CMC-G and CNC components and their percentage in the composites. The theoretic spectra of the composites are similar to CMC-G component spectrum and the second derivatives reveal no new bands, only a slight difference in the intensity of the CMC-G bands. On the contrary, the experimental derivative spectra show differences in the bands position and intensities, indicating interactions by H-bonds.

**Figure 3:**
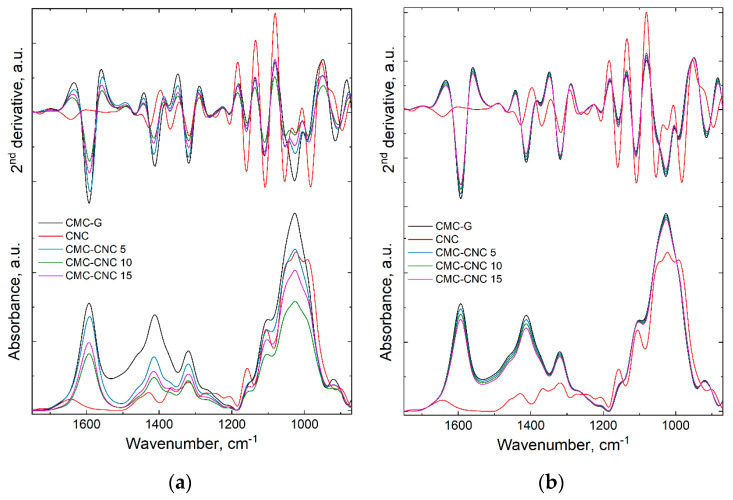
Experimental (**a**) and theoretic (**b**) infrared spectra and their second derivatives in the 1750–870 cm^−1^ region of the components and nanocomposites.

The second region (1750–870 cm^−1^) is assigned to the different stretching and bending vibrations of the functional groups from the CMC-G and CNC structures, and it is presented in Figure 3a (experimental) and 3b (theoretic). In this region, there is a strong difference between the experimental (Figure 3a) and theoretic (Figure 3b) spectra, which may indicate the presence of interactions between the CMC-G and CNC. The theoretic spectra of the composite materials are similar to the CMC-G spectrum, while the experimental spectra of the composite films present differences in bands intensities or maxima. There is a strong decrease of the band from 1590 cm^−1^ assigned to the antisymmetric stretching vibration of COO- groups in the CMC-G component [23,24] and a shifting to a higher wavenumber of the band from 1511 cm^−1^ (in CMC-G) to 1517 cm^−1^ (in CMC-CNC-15), indicating the presence of interactions which change the environment of the C=C groups. It is well known that the shifting to a higher or lower wavenumber of a certain band indicates changes in the surrounding environment of the groups, which adsorb at that certain frequency.

Furthermore, the presence of interactions is indicated by the slight increase and shift to lower wavenumber of the band from 1374 cm^−1^ (in CMC-G spectrum) to 1369 cm^−1^ (in CMC-CNC 15 and CNC spectra). This band is assigned to C–H deformation vibration in cellulose [22,23,24]. The bands from 1412 and 1319 cm^−1^ present a stronger decrease in intensity in experimental spectra, compared to theoretic ones. These bands are assigned to C–H and O–H deformation vibration in both CMC-G and CNC components [22,23,24]. Other visible modifications can be observed for the bands from 1159 to 1109 cm^−1^ present in both components and the band from 1053 cm^−1^ observable in the CNC spectrum. These bands are assigned to C–O and C–O–C stretching vibration [22,23,24].

Due to similarities between the components, in order to highlight the small differences appearing in the spectra and identify the presence of interactions, more sensitive methods (principal component analysis and two-dimensional correlation spectroscopy (2D-COS)) were used.

By *principal component analysis*, it was possible to observe the relations between different independent variables and the differences between spectra and the parameters that describe the interactions between the CMC-G and CNC components.

**Figure 4:**
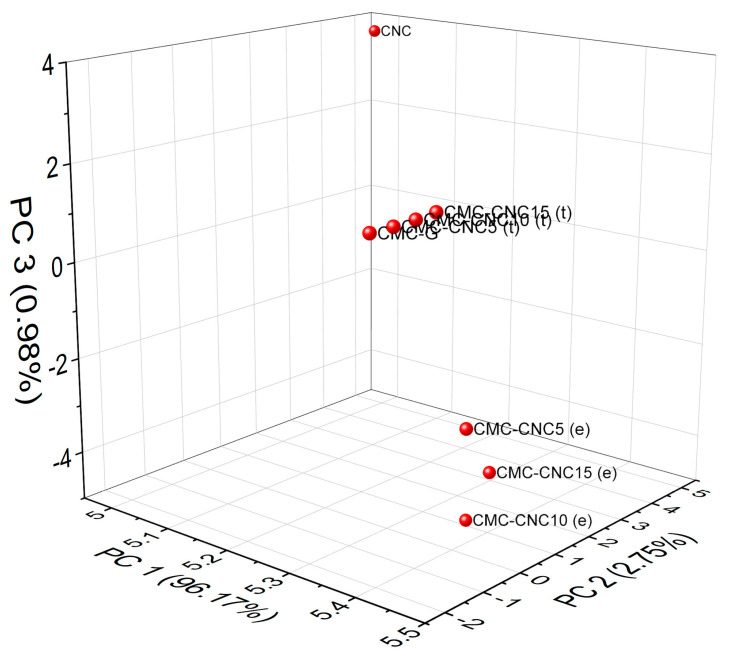
PC scores plot of the experimental and theoretic data.

PC1 (principal component 1) describes 96.17%, PC2 describes 2.75%, and PC3 describes 0.98%. The plotting of the three PC scores (Figure 4) show that the theoretic spectra are in the same plane with the CMC-G component, the plane of PC1–PC2, while the CNC and the composite spectra are separated, presenting variation on the PC3 score direction. The PC1–PC2 plane can be considered the reference (no interactions) plane, while the other planes can be defined as interaction planes. 

**Figure 5:**
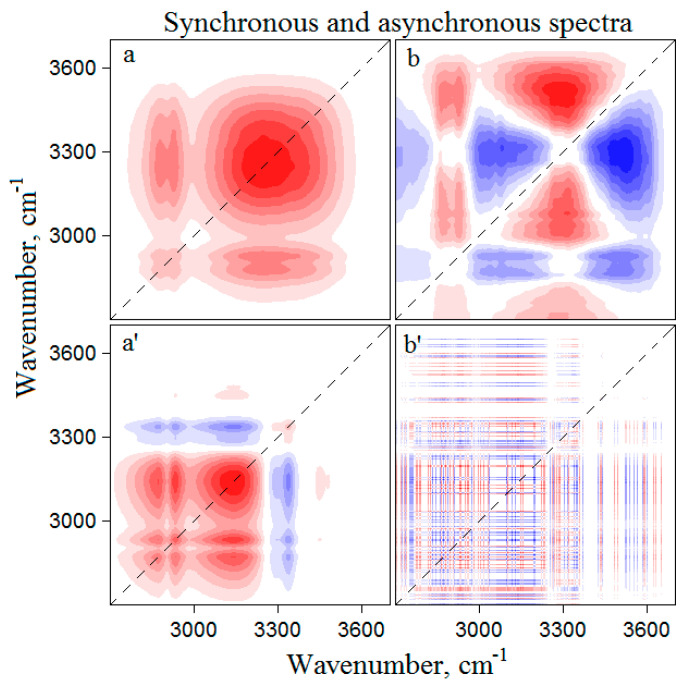
Experimental (**a**) synchronous and (**b**) asynchronous spectra and theoretical (**a’**) synchronous and (**b’**) asynchronous spectra in the 3720–2700 cm^−1^ region.

**Figure 6:**
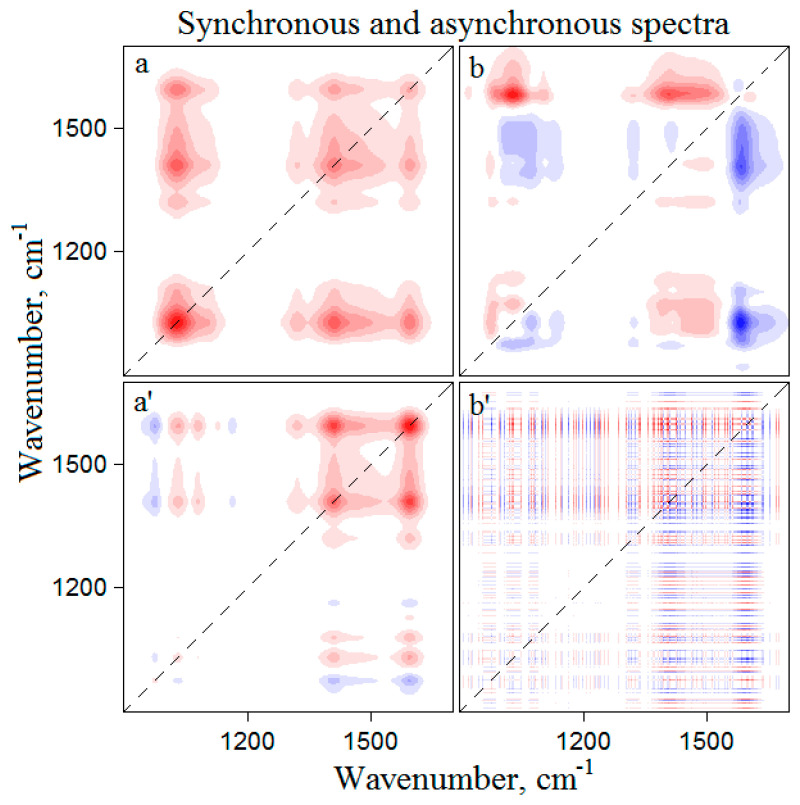
Experimental (**a**) synchronous and (**b**) asynchronous spectra and theoretical (**a’**) synchronous and (**b’**) asynchronous spectra in the 1750–870 cm^−1^ region.

*2D-COS (two-dimensional correlation spectroscopy)* was developed by Noda [25] and further used by many researchers. It is a sensitive technique that highlights the modifications appearing in the structure of the studied components when an external perturbation is present (i.e., variations in concentration). The 2D-COS maps (synchronous and asynchronous ones) for the composite materials in the two regions are presented in Figure 5 and Figure 6.

In the case of no interactions (immiscible components), usually, the synchronous spectrum can be calculated, but not the asynchronous one (examples are Figure 5a’,b’ and Figure 6a’,b’). If between the components some interactions are present, they will cause variations in the intensity and width of certain bands, as well as shifting of the maxima to a higher or lower wavenumber. In our case, for the experimental spectra, both synchronous and asynchronous spectra were obtained, indicating interactions taking place between the components.

The synchronous spectrum of theoretic data (Figure 5a’) in the 3720–2700 cm^−1^ region show four auto-peaks at 3334, 3139, 2925, and 2863 cm^−1^, three positive cross-peaks at 3139–2925 cm^−1^, 3139–2863 cm^−1^, and 2925–2863 cm^−1^ and three negative cross-peaks at 3334–3139 cm^−1^, 3334–2925 cm^−1^, and 3334–2863 cm^−1^. The positive cross-peaks indicate that the bands involved increase or decrease together, while the negative ones indicate that one intensity increases while the other one decreases.

For the experimental spectra, the synchronous spectrum (Figure 5a) present three auto-peaks at 3368, 2921, and 2878 cm^−1^. All the cross-peaks formed have positive values, indicating that all the bands vary in the same direction. The asynchronous spectrum (Figure 5b) shows clear peaks, indicating the specific interaction that takes place between the composite components. From here were identified bands at 3522, 3482, 3295, 3085, and 3030 cm^−1^, and they can be considered as interaction bands with the formation of different H-bonds between the CMC-G and CNC hydroxyl groups.

The next region (1750–870 cm^−1^) in the synchronous spectrum of the theoretic data (Figure 6a’) shows two auto-peaks at 1590 and 1409 cm^−1^ and a series of positive and negative cross-peaks. In case of the experimental data, the synchronous spectrum (Figure 6a) presents three auto-peaks at 1585, 1412, and 1030 cm^−1^, which form only positive cross-peaks. Comparing to the theoretical spectrum, in the experimental one, there is a new auto-peak at 1030 cm^−1^ assigned to the C–O stretching vibration. The asynchronous spectrum of the experimental data (Figure 6b) presents several positive and negative cross-peaks formed by bands from 1585, 1481, 1407, 1318, 1104, 1073, and 1030 cm^−1^. This indicates the involvement of C–O, C–O–C, and CH groups in the interactions or modifications in their surrounding environment.

### 3.2. X-Ray Diffraction

**Figure 7:**
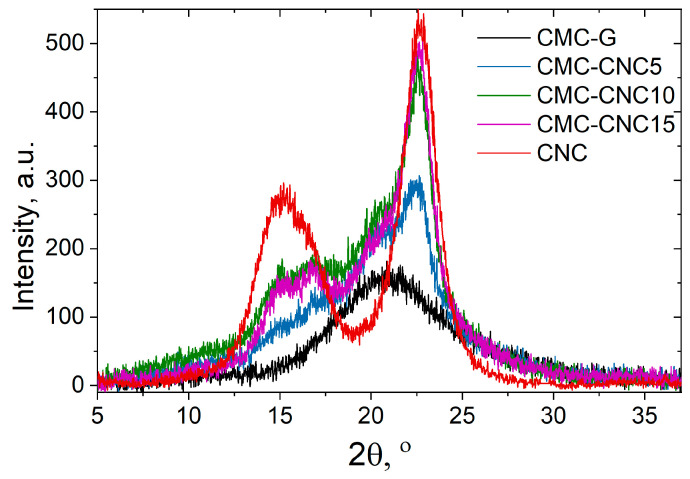
XRD pattern of the components and the nanocomposites.

The diffractograms of the studied samples are presented in Figure 7. The XRD diffraction pattern of the CMC does not show any crystalline peak, only a large signal at 20.7°. A similar pattern was observed by Gupta et al. [26]. On the other side, CNC present the similar diffraction patterns to cellulose I with signals at 15.2°, 16.4°, 20.4°, and 22.6° assigned to (1–10), (110), (102), and (200) crystallographic planes [15,16,22,27,28]. Both 16.4° and 20.4° peaks are merged with the other two peaks from 15.2° and 22.6°, being almost unidentifiable.

After the addition of CNC into the CMC matrix, the peaks from 15.2° and 16.9° are more visible, while the peak from 20.6° presents higher intensity, being a combination of the CMC and CNC peaks, which are situated in about the same place. All the CNC signals increase with the increase of the CNC content in the nanocomposites. 

The separation of the two peaks from 15.2° and 16.9° and the shifting of the last one from 16.4° (in CNC) to 16.9° (in nanocomposites) may indicate interactions taking place between the CMC and CNC, resulting in a disruption or rearrangement in the regular orientation of the CNC crystallites after the addition of the amorphous matrix.

The crystallinity degree, the crystallite sizes and d-spacing are presented in Table 2.

The crystallinity of the CNC was of about 73%, while in the nanocomposites, the maximum value was obtained for the CMC-CNC15, due to a higher amount of the CNC into the composite material. The crystallite size was 4.02 for CNC and increased to 5.20, 5.33 and to 5.39 for the nanocomposites, while the d-spacing was of 0.387 for pure CNC and slightly increased for the nanocomposites, the highest value being observed for the CMC-CNC5 formulation. This increase might be due to the attachment of the amorphous matrix on the surface of the crystallites.

### 3.3. Scanning Electron Microscopy (SEM)

**Figure 8:**
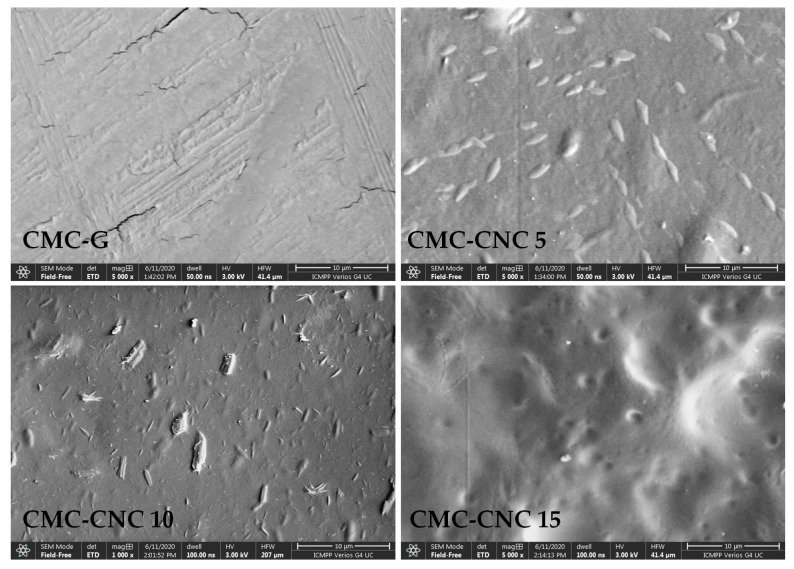
Morphological aspect of CMC and CMC-CNC nanocomposite films.

The scanning electron microscopy (SEM) images of the CMC-G and CMC- CNC nanocomposite films are presented in Figure 8.

The surface of the CMC film shows a uniform and smooth surface with small cracks being a sign of a highly ordered morphology of the matrix. The addition of the CNCs into the biopolymeric matrix led to a surface that was less homogeneous, and the CNCs distribution is well dispersed. The occurred changes at the surface of samples confirmed the compatibility between the CMC and the reinforcing agent due to the high number of hydrogen bonds between the components [22].

### 3.4. Water Vapor Sorption Properties

The water vapor sorption properties of the nanocomposite films were evaluated in order to identify the influence of CNC nanofiller on the structural arrangements, pores size and number, and water molecules’ ability to penetrate and link to a polymeric film structure.

Both CMC and CNC are hydrophilic materials; i.e., they adsorb water molecules from the surrounding environment by contact with water, due to the high number of hydroxyl groups in their structure, which make them sensitive to water adsorption [29,30].

Van der Well et al. [31] described the water adsorption as being a process in which the molecules of water are adsorbed by the material through “physical adsorption”, “chemisorption”, or/and “multilayer condensation”. Moreover, they mentioned the existence of three regions: I—up to 40% RH where single molecules with weak interactions are present, II—aggregated water molecules that interact, forming aggregates or clusters, and III—specific to localized interactions of the water molecules with polymeric substrate.

**Figure 9:**
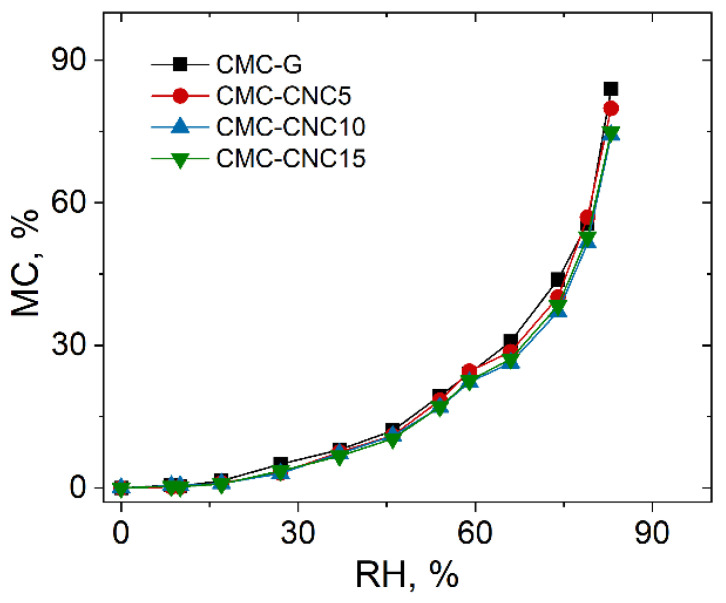
Sorption isotherms for the studied samples.

In our case, all the sorption isotherms of the studied materials (Figure 9) present the IUPAC Type III isotherms describing the adsorption of molecules on microporous and non-porous solids which are less hydrophilic and with weak adsorbate–adsorbent interactions [31]. The characteristic shape of the isotherm shows low moisture contents (less than 5% MC) at low relative humidity (up to about 30% RH) and the uptake gradually increases at high relative humidity values, presenting an asymptotic curve. All the sorption isotherms presented similar values of the MCs up to about 30% RH. After this value, the samples that have CNC in their composition recorded lower MC values compared to the CMC-G sample. Moreover, this value decreased with the increase of the CNC content.

The reduction in the amount of adsorbed water into the composite matrix may be due to the decrease of the available hydroxyl groups from the components due to their participation in intermolecular interactions, which tend to stabilize the polymeric matrix. Similar results were obtained by Lu et al. when they added CNC into plasticized starch [32]. In addition, Yin et al. observed that bacterial cellulose nanocrystals added in natural rubber decreased the storage modulus of films after equilibrium swelling in water due to the disentanglement of the hydrophilic filler network via competitive hydrogen bonding with water [33]. On the other hand, Mutjaba et al. observed that in the case of chitosan films reinforced with CNC, there was no significant improvement in water vapor permeability due to the overall lower film crystallinity [34].

**Figure 10:**
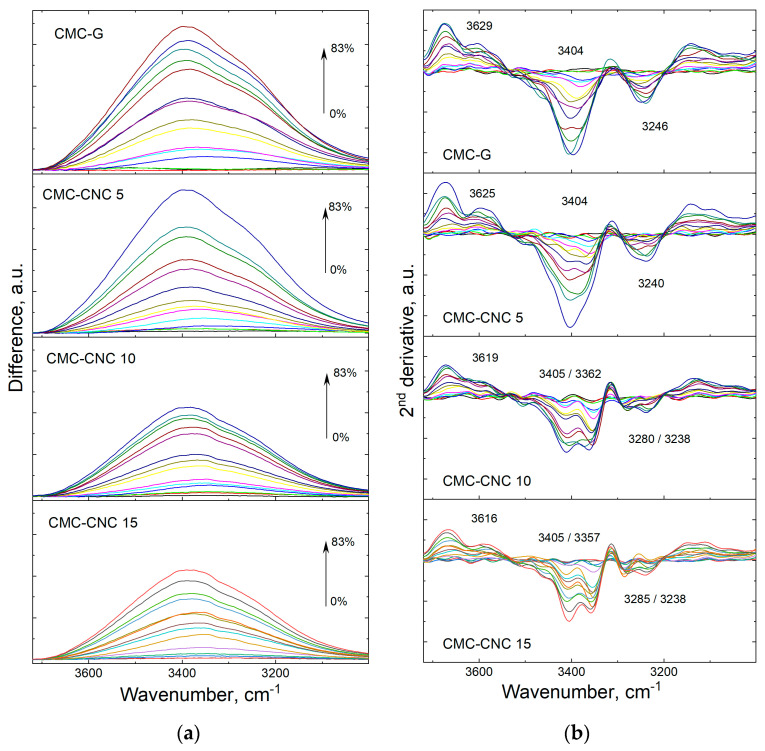
Difference infrared spectra (**a**) showing the increase of the integral area of the water band with the increase in MC content and their second derivatives (**b**).

Infrared spectroscopy was involved in order to try to identify the dependence of the integral areas of the water bands with the moisture content and also to identify the possible interactions that may take place between the water molecules and between the water molecules and OH groups from the composite matrix. The water adsorption into the films can be identified by strong variations of the bands from 3720 to 3000 cm^−1^, which are bands that are associated with the stretching vibration of the OH groups involved in hydrogen bonds. Since other types of H-bonds from the components structure can be found in this region, the difference spectra were calculated by subtracting the spectrum of dried film from all the other spectra (Figure 10a).

The OH stretching vibration band presents a complex profile with one maximum at about 3390 cm^−1^ and a shoulder at about 3265 cm^−1^, suggesting the presence of at least two different H-bond interactions.

In the literature [30,31,35], it has been mentioned that the absorbed water molecules are present in three different states: (1) free/single water molecules; (2) aggregated water molecules or presenting localized (weak) interactions with the polymeric matrix and (3) clustering or presenting strong interactions with the polymeric matrix. All these have distinct thermodynamic properties which depend on the type or degree of interaction and the polymeric structure.

Cotugno et al. [36] indicated that the three different states can be spectroscopically distinguished: (A0) assigned to asymmetric OH stretching of non-associated water, representing the water molecules that do not form any hydrogen bond with the polymer matrix, i.e., (1); (A1) assigned to water molecules bonded to specific sites via weak hydrogen bond interactions, i.e., (2); and (A2) assigned to water molecules bonded to specific sites via strong hydrogen bond interactions, i.e., (3) [22,30,36].

When the second derivatives of the difference band were performed (Figure 10b), they showed the presence of three distinct signals for CMC-G at 3629, 3404, and 3246 cm^−1^. The three bands are assigned to A0, A1, and A2, respectively. After adding the CNC into the polymeric matrix, together with the bands from 3404 and 3246 cm^−1^, two separate maxima at about 3362 and 3285 cm^−1^ were identified. The later two bands increase in intensity with the increase in the amount of CNC; therefore, they might suggest that the position of the bands assigned to the stretching vibration of the water molecules bonded to CMC and CNC are different. In other words, the bands from 3404 and 3246 cm^−1^ are assigned to water molecules bonded to the CMC matrix, while the bands from 3362 and 3285 cm^−1^ are assigned to water molecules bonded to CNC.

**Figure 11:**
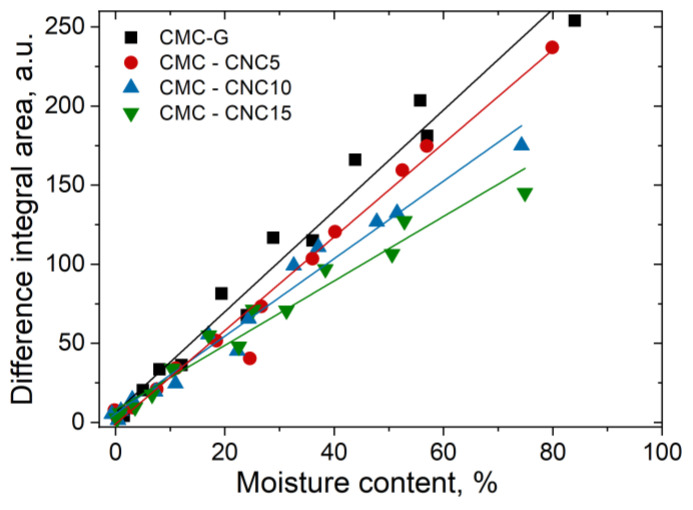
Integral area of the difference bands vs. moisture content.

The difference bands (Figure 10a) show the increase of the integral areas as a function of the increase of moisture content in the samples, as well as a decrease in the integral area with the increase of the CNC content in the composites. At the same time, the integral areas present a linear dependence with the MC content (Figure 11).

The same behaviors were observed when other components were used as polymeric matrices, i.e., poly vinyl alcohol (PVA)/starch (S) [30] or κ-carrageenan [22].

### 3.5. Water Uptake

The CMC-G and the composite films were exposed in a closed container with an average relative humidity of 57% and weighted every hour until no increase in the mass of the samples was observed.

**Figure 12:**
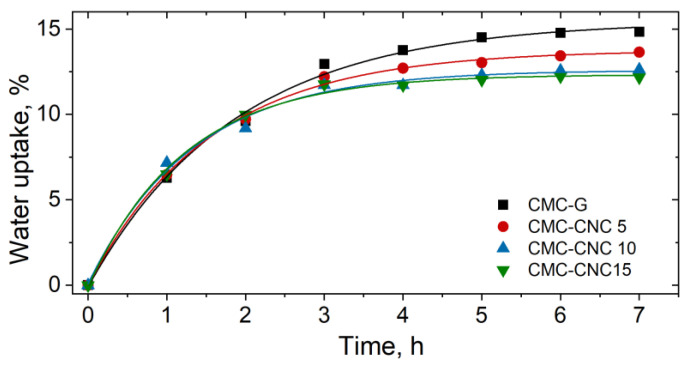
Water uptake recorded as a function of time for the studied samples.

The percentage of the water uptake for the studied films is presented in Figure 12. As can be observed, after about 6 h, all the samples reached equilibrium. The highest amount of adsorbed water was recorded for the CMC-G film of 14.8%, while the other films recorded values of 13.6% (CMC-CNC 5), 12.6% (CMC-CNC 10), and 12.1% (CMC-CNC 15), respectively.

**Scheme 1:**
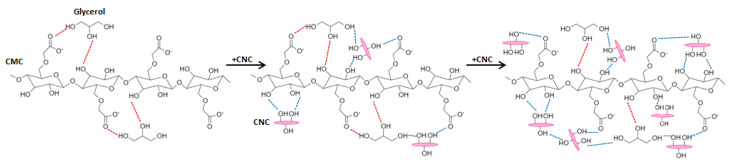
Representation of possible interactions between CMC, G, and CNC.

Similar behaviors were observed when other polymeric matrices were used in combination with CNC [22,30], proving once again that the filler is interacting with the matrix; therefore, the number of available OH groups (the main sorption sites) is reduced (Scheme 1).

## 4. Conclusions

In this study, various amounts of CNCs used as nanofiller were incorporated into the CMC-G matrix in order to obtain nanocomposite systems with improved properties. The addition of CNC induced structural changes by the presence of interactions between the components, forming a more compact and stable film. The increase in CNC concentration into the CMC-G matrix induced a reduction in the amount of water content adsorbed. This behavior is due to the presence of the interactions that takes place, reducing therefore the sorption sites available for the water molecules. Infrared spectroscopy also revealed different adsorption bands for the water molecules, presenting weak or strong interactions with the CNC and CMC polymers. The amount of adsorbed water reached an equilibrium after about 6 h at 57% RH.

## Figures and Tables

**Table 1:** Samples composition and codes.

Codes	Composition		
	CMC	CNC	G ^(⁂)^
CMC-G	100	-	30
CNC	-	100	-
CMC-CNC 5	95	5	30
CMC-CNC 10	90	10	30
CMC-CNC 15	85	15	30

^(⁂)^ the percentage of G is % from the total mass of CMC + CNC.

**Table 2:** Crystallinity degree, the crystallite sizes and d-spacing for the (200) crystallographic plane.

Codes	CNC	CMC-CNC 15	CMC-CNC 10	CMC-CNC 5
Cr.I %	73.3	44.8	38.0	28.9
τ_200_	4.02 ± 0.01	5.20 ± 0.02	5.33 ± 0.01	5.39 ± 0.02
d_200_	0.387	0.388	0.388	0.390
